# Knowledge, Attitude, and Practice regarding Food, and Waterborne Outbreak after Massive Diarrhea Outbreak in Yazd Province, Iran, Summer 2013

**DOI:** 10.1155/2014/403058

**Published:** 2014-10-30

**Authors:** Zahra Cheraghi, Batul Okhovat, Amin Doosti Irani, Mojgan Talaei, Elham Ahmadnezhad, Mohammad Mehdi Gooya, Mahmood Soroush, Hossein Masoumi Asl, Kourosh Holakouie-Naieni

**Affiliations:** ^1^Department of Epidemiology & Biostatistics, School of Public Health, Tehran University of Medical Sciences, Tehran 6446-14155, Iran; ^2^Department of Health in Emergencies & Disasters, National Institute of Health Research, Tehran University of Medical Sciences, Iran; ^3^Center of Disease Control (CDC), Ministry of Health, Tehran, Iran; ^4^Food Microbiology Research Center, Tehran University of Medical Sciences, Tehran 6446-14155, Iran

## Abstract

*Objective*. This Study was conducted after a diarrhea outbreak that occurred in Yazd Province, Iran. The aim of the study was to compare knowledge, attitude, practice, and other risk factors of the affected communities regarding diarrhea outbreak (the cities of Zarch, Meybod, and Ardakan) to nonaffected communities (the cities of Yazd and Taft). *Methods*. A knowledge, attitude, and practice (KAP) survey study was conducted from August to September 2013 enrolling 505 subjects who were referred to health centers anonymously during the epidemic. The questionnaire included the following four parts: (a) general characteristics such as gender, education level, source of health information obtaining; (b) 12 questions on knowledge (Min = 0, Max = 36); (c) 10 questions on attitude (Min = 0, Max = 50); and (d) nine questions on practice (Min = 0, Max = 27). *Results*. The overall mean score of knowledge, attitude, and practice was 28.17 (SD = 4.58), 37.07 (SD = 4.39), and 21.31 (SD = 3.81), respectively. Practice on food- and waterborne outbreaks was significantly higher in females (*P* = 0.001) and in nonaffected communities (*P* = 0.031). *Conclusions*. Nonaffected communities had a considerably better practice score. With the increase in the score of knowledge about food- and waterborne outbreaks, the score of practice increased slightly.

## 1. Introduction 

Food- and waterborne illnesses are a growing public health problem worldwide. Food contaminated with microorganisms such as parasites, microbes, and other pathogens is the main cause [1]. The most virulent pathogens causing food- and waterborne diseases are campylobacter, Escherichia coli, salmonella, and Shigella [2]. According to the Center for Disease Control, a food- and waterborne disease outbreak occurs when two or more people have the same disease from a common contaminated food or drink source [3]. The prevalent symptoms include an upset stomach, abdominal cramps, vomiting, diarrhea, fever, and dehydration [4]. According to the WHO, “millions of people become ill and thousands die from preventable food- and waterborne diseases annually” [5]. About 48 million Americans become sick due to contaminated food and water each year [3]. Shigellosis is responsible for 80 million cases of dysentery and 700,000 deaths due to dysentery in the world [6]. WHO suggests several preventive keys for safe food, including keeping the food clean, separating raw and cooked foods, keeping food at safe temperatures, and using safe water and raw materials [5]. Most studies have indicated that the knowledge about food- and waterborne outbreaks is low especially in young age groups [7–10].

The knowledge, attitude, and practice (KAP) studies are one of the best ways of assessing knowledge, attitude, and practice of individuals [11]. The current study was conducted during and following the diarrhea outbreak in the cities of Zarach, Ardakan, and Meybod located in Yazd on August 9, 2013. Also, most of the cases were reported from Meybod. The overall incidence rate was 13.19 per 1000,000. A case of diarrhea was defined as an episode with three or more watery stools over a period of 24 hours, with or without other symptoms during the outbreak period [12]. One of the main reasons for conducting this study was to compare knowledge, attitude, and practice of affected communities (the cities of Zarach, Meybod, and Ardakan) and nonaffected communities (the cities of Yazd and Taft).

## 2. Materials and Methods

### 2.1. Study Design

This was a cross-sectional survey. Knowledge, attitude, and practice (KAP) study was conducted from August to September 2013 in Yazd Province, which is located in the center of Iran. All participants were enrolled voluntarily and anonymously in the study.

### 2.2. Study Area

**Figure 1:**
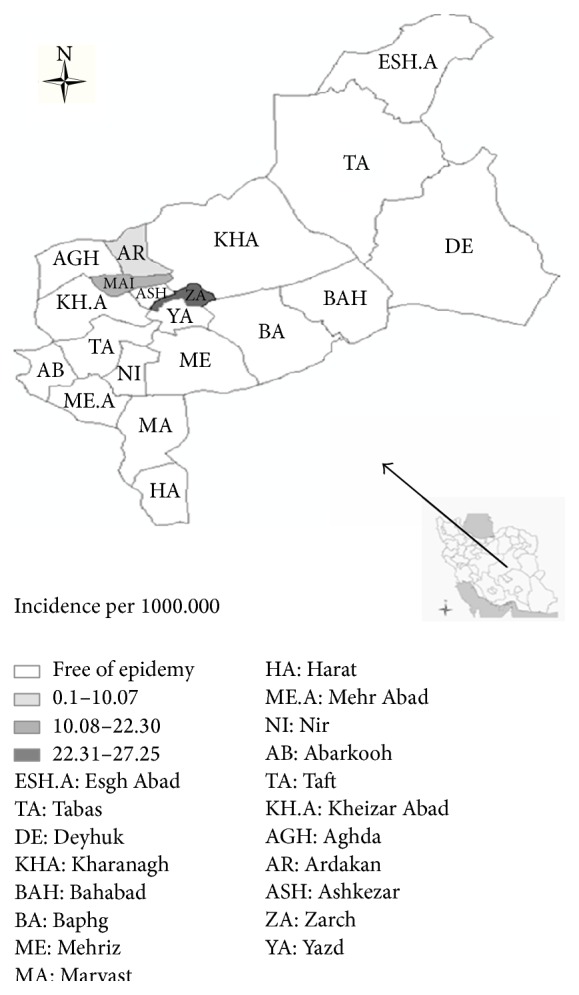
Incidence rate of food- and waterborne outbreak in Yazd province (Iran) in summer 2013.

The study was conducted in Yazd Province. Yazd Province, with an area of 131,551 km^2^ and a population of 972,781 people, is located as an oasis in the Dasht-e Kavir desert. Yazd Province is divided into 9 cities including Abarkuh, Ardakan, Bafq, Khatam, Meybod, Mehriz, Sadough, Taft, and Yazd. We conducted our study in the cities of Zarach, Meybod, Ardakan, Yazd, and Taft (see Figure 1).

### 2.3. Sampling and Sample Size

Our study encompassed 505 people aged over 15 years who were visited at different health centers in Yazd Province from 27 August to 10 September 2013 during the epidemic. We conducted this survey during the diarrhea outbreak. Between August 9 and September 27, 2013, a diarrhea outbreak occurred in the cities of Meybod, Ardakan and Zarach. We considered these cities as affected cite and the neighboring cites (Yazd and Taft) were considered as nonaffected cites. Also, theses cites were the nearest cities to Meybod, Ardakan and Zarach.

The sample size was calculated based on a pilot study. In the pilot study, the prevalence of weak practice regarding food- and waterborne diseases was estimated to be 27%. Assuming the prevalence of weak practice regarding food- and waterborne diseases, the sample size was 259 with the significance level set at 0.05. Because we had two communities (Yazd and Taft as nonaffected communities of diarrhea outbreak and Meybod, Ardakan, and Zarach as affected communities), we increased the sample size to 518. The response rate was 86.91%.

### 2.4. Data Gathering

The questionnaire consisted of four sections as follows: (a) 18 questions on general characteristics such as gender, education level and history of severe diarrhea in the past 30 days, and the main source of health information obtaining in the past 30 days; (b) 12 questions related to knowledge of food- and waterborne diseases including three-choice questions (Yes/No/Do not know) and five-choice questions (A/B/C/D/Do not know), with a total score between zero and 36; (c) 10 five-choice questions (Strongly Agree/Agree/No Idea/Disagree/Strongly Disagree) related to attitude toward food- and waterborne diseases, with a total score between zero to 50; and (d) nine questions related to practice on food- and waterborne diseases, with a total score between zero to 27.

We considered high knowledge, positive attitude, and good practice if participant answered ≥%75 of the questions correctly, moderate knowledge, attitude, and practice if they answered 60–74% of the questions correctly, and low knowledge, negative attitude, and weak practice if they answered <60% of the questions correctly.

The reliability of the questionnaire was investigated by conducting a pilot study on 30 people. The value of Cornbrash's alpha coefficient for the knowledge and attitude questions was 0.60. Also, the sample size calculated according to a pilot study, bases on the weak practice proportion (0.27).

### 2.5. Statistical Analysis

Analysis of variance was used to compare the mean score of knowledge, attitude, and practice across subgroups. An adjusted linear regression model was employed to estimate the effect of knowledge, attitude, and other related factors on practice regarding food- and waterborne outbreaks. All analyses were performed at the 5% significance level (*P* < 0.05) using Stata 12 (StataCorp, College Station, TX, USA).

## 3. Results

From 505 subjects enrolled in the study, 54.46% (275) were female and 45.54% (230) were male. The mean age of the participants was 32.35 ± 0.48 years.

The total mean score of the participants' knowledge, attitude, and practice regarding food- and waterborne diseases was 28.17 (SD = 4.58), 37.07 (SD = 4.39), and 21.17 (SD = 3.81), respectively. Sixty-four percent of the participants had high knowledge and good practice regarding food- and waterborne outbreaks and 43% of them had a positive attitude.

### 3.1. Mean Difference of KAP by Demographic and Prognostic Factors (Table 1)

Nonaffected communities by the diarrhea outbreak (Yazd and Taft cites) had high mean scores of knowledge, attitude, and practice in comparison with the affected communities (Meybod, Ardakan and Zarach). However, only the difference in the mean score of knowledge was significant (*P* = 0.037). The mean of knowledge and practice scores in females was high (28.73 SD = 4.13, 21.77 SD = 3.75, resp.) in comparison with males (*P* = 0.001). Married participants had high mean scores of knowledge and practice (28.0 SD = 1.41, 22.0 SD = 1.41) in comparison with singles (*P* = 0.004, *P* = 0.019, resp.). The maximum knowledge was observed in >60-year-old participants (29.87, SD = 3.18, *P* < 0.001), and the maximum mean of attitude and practice was seen in participants aged 25–40 years (*P* = 0.057, *P* = 0.240), too.

The housekeeper's knowledge and practice were higher in comparison with other family members (*P* = 0.001, *P* = 0.015) but householders had a better attitude (*P* = 0.042). Participants reported television and health centers as the main sources of obtaining health information in the past 30 days (38% and 31.27%, resp.). The mean scores of knowledge and practice among people who reported health centers as the main sources of obtaining health information were high (*P* = 0.011 and *P* = 0.105, resp.). The maximum scores of knowledge and attitude were in 4–6-person families (*P* = 0.053 and *P* = 0.012, resp.). The mean scores of knowledge, attitude, and practice of people who had severe diarrhea in the past 30 days were low (*P* = 0.01, *P* = 0.029, and *P* = 0.168, resp.) (See Table 1).

### 3.2. Adjusted Linear Regression Analysis Result (Table 2)

According to adjusted linear regression analysis, practice regarding food- and waterborne diseases increased 0.08% per one-unit increase in the score of knowledge (*P* < 0.001) and 0.16% per one-unit increase in the score of attitude (*P* < 0.001). Knowledge increased 0.10% per one-year increase in age (*P* < 0.001) (see Table 2). Also, people who had academic education had 6.01 times better practice regarding foodborne and waterborne outbreaks in comparison with illiterate participants (*P* < 0.001) (See Table 2).

## 4. Discussion

The total mean scores of knowledge, attitude, and practice based on primary categorization were acceptable. Our result indicated that the average of KAP was low in affected communities (Zarach, Meybod, and Aradakan). The low level of KAP can be a predisposing factor (condition) to a diarrhea outbreak. The total mean scores of KAP were high in females. This finding is also reported in several studies [9, 10, 13] and is corresponding to this finding of our study that the mean scores of knowledge and practice were highest among housekeepers (since the majority of housekeeper are female), too. One of the possible reasons for this may be the fact that housewives spent more time obtaining health information from sources such as television and health centers, so that obligatory educational program could be improving the level of knowledge of men. The result of analysis of variance showed that with the increase in the level of education in men, the mean score of KAP increases but the maximum mean of KAP scores was observed in illiterate most justifiable reason result in low sample size (*n* = 9) in illiterate group and subsequently the probability of random error occurrence.

Framers had best practice against food- and waterborne outbreaks in comparison with other jobs but this statistically significant relation may be not true, because of very low sample (*n* = 6) in farmers category and consequently increasing probability of selection bias occurrence.

The maximum mean scores of knowledge, attitude, and practice were observed among people who reported health centers as the main sources of obtaining health information in the past 30 days. On the other hand, 38% (maximum value) of the people with high knowledge reported health centers as main sources of obtaining health information. This finding indicates the effective role of health centers in increasing the level of knowledge.

The mean scores of knowledge, attitude, and practice of people who had severe diarrhea in the past 30 days were low. These mean differences may introduce that, with the low level of knowledge, negative attitude, and weak practice, people become more susceptible to get food- and waterborne diseases as a hypothesis. Thus this finding indicated KAP study may be useful tool for identifying high risk group who gets food- and waterborne disease.

Mpazi and Mnyika [8] reported that 83% of the women and 87% of the men had high knowledge about foodborne diseases, with nonsignificant relation with high response rate (84.4%). In our study, the response rate was high but 69% of the women and 56% of the men had good knowledge. This difference may be due to different categorization of knowledge in the design of the studies. Another study conducted by Saleeon [14] showed that 57%, 48%, and 89% had high mean scores of knowledge, attitude, and practice, respectively. In our study, the mentioned scores were 63.17%, 39.21%, and 63.96%, respectively. In both studies, about 75–82% referred to the fact that they always boiled canned food for 20–30 minutes before consuming it. About 99% of the participants in a study by Askarian et al. [15] and 70% of the participants in our study believed that they had to separate raw and cooked foods. In a study by Unusan [16], participants knew nausea (69%), fever (6.1%), and diarrhea (5%) as the symptoms of food- and waterborne diseases while in our study, 14%, 81.91%, and 63.76% of the participants mentioned these symptoms, respectively. We used free-choice optioned from this question these, maybe justified differences. Norazmir et al. [17] reported a weak positive correlation between food safety knowledge and practices regarding food safety; in our study, this correlation was even weaker.

*Limitations*. In our study, the rejection rate was 13.09, which could decrease generalizability. Our study subjects were those who were visited at health centers, which may cause selection bias although we tried to decrease this bias with sampling from two big reference health centers located in different parts of the cities. Another limitation that should be noted was the relatively low reliability (Chronbach's alpha = 0.6) of the questionnaire, which may increase the information bias.

## 5. Conclusion

Nonaffected communities by the diarrhea outbreak had a considerably better practice score. With the increase in the score of knowledge about food and water outbreak, the score of practice increased slightly.

## Figures and Tables

**Table 1:** Mean score of knowledge, attitude, and practice of the participants regarding water- and foodborne outbreaks by demographic characteristics using analysis of variance method.

Variables		Number	Percent	Knowledge			Attitude			Practice		
				Mean	SD	*P* value	Mean	SD	*P* value	Mean	SD	*P* value
Location	Affected cities	248	49.11	27.80	4.34	**0.037**	36.84	4.32	0.127	21.04	3.87	0.057
	Nonaffected cities	257	50.89	28.53	4.78		37.29	4.46		21.57	3.75	

Sex	Male	230	45.54	27.51	4.99	**0.001**	36.9	4.04	0.209	20.76	3.82	**0.001**
	Female	275	54.46	28.73	4.13		37.21	4.66		21.77	3.75	

Marital status	Married	403	80.12	28.51	4.52	**0.004**	37.14	4.46	0.219	21.50	3.68	**0.019**
	Single	100	19.88	26.80	4.36		37.76	4.12		20.53	4.27	

Age group	15–24 years	115	23.57	26.62	4.33	**<0.001**	36.40	4.08	**0.057**	20.67	4.04	0.243
	25–40 years	269	55.12	28.60	4.49		37.58	4.21		21.54	3.80	
	41–60 years	96	19.67	29.25	4.31		36.90	4.82		21.37	3.76	
	>60 years	8	1.64	29.87	3.18		35.50	4.82		21.50	1.92′	

Household number	1–3 people	204	42.68	28.25	4.58	0.053	36.78	4.38	**0.012**	21.07	3.82	0.407
	4–6 people	251	25.51	28.37	4.51		37.44	4.40		21.51	3.83	
	>7 people	23	4.81	25.92	5.30		34.82	3.44		20.86	4.19	

Education	Illiterate	9	1.79	30.33	2.82	**0.0002**	36.00	4.87	**<0.001**	16.66	4.55	**0.002**
	Primary school	31	6.16	27.22	5.09		35.87	4.08		21.86	3.14	
	**Secondary school**	61	12.13	26.95	4.76		34.68	4.16		21.32	3.85	
	High School	162	32.21	27.37	4.09		36.47	4.22		21.21	3.76	
	Academic	240	71.71	29.07	4.67		38.25	4.24		21.49	3.80	

Membership in family	Householder	178	35.39	28.07	4.65	**<0.001**	37.27	3.83	**0.042**	21.26	3.50	**0.015**
	Housekeeper	202	40.16	28.52	4.35		36.91	4.70		21.63	3.83	
	Offspring	94	18.69	26.67	4.89		36.40	4.22		20.34	3.83	
	Other	29	5.77	31.24	2.82		38.96	5.49		20.34	4.12	

Job	Pupil	15	2.99	27.26	3.23	**<0.001**	36.20	4.00	**0.0001**	20.00	3.48	**0.043**
	Collegian	59	11.78	26.84	5.25		37.42	4.34		20.84	4.00	
	Employee	136	27.15	29.94	4.15		38.68	4.33		21.65	3.80	
	Worker	74	14.77	26.25	4.74		36.04	3.70		20.70	3.80	
	Housekeeper	153	30.54	28.11	4.16		36.23	4.63		21.86	3.73	
	Farmer	6	1.20	26.00	4.00		35.50	4.92		23.50	2.42	
	Retired	4	0.80	30.00	6.87		37.00	3.74		21.50	4.85	
	Other	54	10.78	28.53	4.41		36.83	3.93		20.33	3.88	

Health source information	Health Centers	81	31.27	29.80	4.50	**0.011**	38.09	4.78	**0.003**	22.45	3.44	0.105
	Radio	98	37.84	27.91	4.37		35.00	4.78		21.66	2.77	
	Television	12	4.63	27.58	7.72		35.85	4.48		21.20	4.26	
	Newspapers	12	4.63	27.33	4.65		38.75	5.01		22.00	4.86	
	Other	56	21.62	27.48	4.65		37.51	4.06		20.78	3.49	

**Table 2:** Adjusted Linear regression analysis assessing the effect of related factors on water- and foodborne outbreak.

Variables	Knowledge			Attitude			Practice		
	Coefficient	95% CI	*P* Value	Coefficient	95% CI	*P* Value	Coefficient	95% CI	*P* Value
Age (year)	0.10	[0.06, 0.13]	**<0.001**	0.0002	[−0.36, 0.37]	0.991	0.03	[0.003, 0.07]	**0.03**
Education									
Illiterate (reference)	—	—	—	—	—	—	—	—	—
Elementary	−1.79	[−4.79, 1.19]	0.239	1.07	[−1.87, 4.02]	0.475	6.47	[3.74, 9.20]	**<0.001**
Middle school	−0.58	[−3.47, 2.37]	0.694	−0.06	[−2.91, 2.77]	0.962	6.51	[3.88, 9.12]	**<0.001**
High school	−0.52	[−3.33, 2.27]	0.640	1.60	[−1.15, 4.35]	0.254	6.09	[3.54, 8.64]	**<0.001**
Academic	0.66	[−2.13, 3.46]	0.640	2.81	[0.06, 555]	0.045	6.04	[3.49, 8.58]	**<0.001**
History of severe diarrhea (Yes/No)	−0.75	[−1.87, 0.36]	0.184	−0.20	[−1.30, 0.99]	0.717	−0.71	[−1.73, 0.30]	0.169
Sex (reference: male)	−1.34	[−2.07, −62]	**<0.001**	−0.15	[−0.87, 0.57]	0.680	−0.86	[−1.53, −0.19]	**0.012**
Knowledge (per one-unit score)	—	—	—	0.25	[0.21, 0.29]	**<0.001**	0.08	[0.00, 0.15]	**<0.001**
Attitude (per one-unit score)	0.34	[0.25, 0.42]	**<0.001**	—	—	—	0.16	[0.81, 0.24]	**<0.001**
Constant	13.09	[8.72, 7.48]	0.229	25.47	[21.59, 29.36]	**<0.001**	5.77	[1.61, 9.9]	**<0.007**

## References

[1] World Health Organization General information related to foodborne disease. http://www.who.int/topics/foodborne_diseases/en/.

[2] WHO Foodborne disease surveillance. http://www.who.int/foodborne_disease/en/index.html.

[3] CDC *Tracking and Reporting Foodborne Disease Outbreaks*.

[4] U.S. National Library of Medicine http://www.nlm.nih.gov/medlineplus/foodborneillness.html.

[5] World Health Organization Prevention of foodborne disease: the five keys to safer food. http://www.who.int/topics/food_safety/flyer_keys_en.pdf?ua=1.

[6] WHO (2005). *Guidelines for the Control of Shigellosis, Including Epidemics due to Shigella Dysenteriae Type 1*.

[7] Angelillo I. F., Viggiani N. M. A., Greco R. M., Rito D. (2001). HACCP and food hygiene in hospitals: knowledge, attitudes, and practices of food-services staff in Calabria, Italy. *Infection Control and Hospital Epidemiology*.

[8] Mpazi V. M., Mnyika K. S. (2005). Knowledge, attitudes and practices regarding cholera outbreaks in Ilala Municipality of Dar Es Salaam Region, Tanzania. *East African Journal of Public Health*.

[9] Siow O. N., Sani N. A. (2011). Assessment of Knowledge, Attitudes and Practices (KAP) among food handlers at residential colleges and canteen regarding food safety. *Sains Malaysiana*.

[10] Zain M. M., Naing N. N. (2002). Sociodemographic characteristics of food handlers and their knowledge, attitude and practice towards food sanitation : a preliminary report. *Southeast Asian Journal of Tropical Medicine and Public Health*.

[11] Kaliyaperumal K. (2004). Guideline for conducting a knowledge, attitude and practice (KAP) study. *AECS Illumination*.

[12] CDC http://www.cdc.gov/healthywater/swimming/rwi/illnesses/diarrhea-swimming.html.

[13] Kibret M., Abera B. (2012). The sanitary conditions of foods service establishments and food safety knowledge and practices of food handers in BAHIR DAR town. *Ethiopian Journal of Health Sciences*.

[14] Saleeon T. (2011). *Knowledge, Attitude, and Practice Toward Clostridium Botulinum Outbreak in Home-Canned Bamboo Shoots at Pakaluang Subdistrict, Ban Luang district, Nan province, Thailand*.

[15] Askarian M., Kabir G., Aminbaig M., Memish Z. A., Jafari P. (2004). Knowledge, attitudes, and practices of food service staff regarding food hygiene in Shiraz, Iran. *Infection Control and Hospital Epidemiology*.

[16] Unusan N. (2007). Consumer food safety knowledge and practices in the home in Turkey. *Food Control*.

[17] Norazmir M. N., Noor Hasyimah M. A., Siti Shafurah A., Siti Sabariah B., Ajau D., Norazlanshah H. (2012). Knowledge and practices on food safety among secondary school students in Johor Bahru, Johor, Malaysia. *Pakistan Journal of Nutrition*.

